# Influence of Surface Position along the Working Range of Conoscopic Holography Sensors on Dimensional Verification of AISI 316 Wire EDM Machined Surfaces

**DOI:** 10.3390/s140304495

**Published:** 2014-03-06

**Authors:** Pedro Fernández, David Blanco, Carlos Rico, Gonzalo Valiño, Sabino Mateos

**Affiliations:** Department of Manufacturing Engineering, University of Oviedo, Campus of Gijón, 33203 Gijón, Spain; E-Mails: pedrofa@uniovi.es (P.F.); jcarlosr@uniovi.es (C.R.); gvr@uniovi.es (G.V.); sabino@uniovi.es (S.M.)

**Keywords:** conoscopic holography, depth of field, non-contact measurement, quality

## Abstract

Conoscopic holography (CH) is a non-contact interferometric technique used for surface digitization which presents several advantages over other optical techniques such as laser triangulation. Among others, the ability for the reconstruction of high-sloped surfaces stands out, and so does its lower dependence on surface optical properties. Nevertheless, similarly to other optical systems, adjustment of CH sensors requires an adequate selection of configuration parameters for ensuring a high quality surface digitizing. This should be done on a surface located as close as possible to the stand-off distance by tuning frequency (*F*) and power (*P*) until the quality indicators Signal-to-Noise Ratio (*SNR*) and signal envelope (*Total*) meet proper values. However, not all the points of an actual surface are located at the stand-off distance, but they could be located throughout the whole working range (WR). Thus, the quality of a digitized surface may not be uniform. The present work analyses how the quality of a reconstructed surface is affected by its relative position within the WR under different combinations of the parameters *F* and *P*. Experiments have been conducted on AISI 316 wire EDM machined flat surfaces. The number of high-quality points digitized as well as distance measurements between different surfaces throughout the WR allowed for comparing the metrological behaviour of the CH sensor with respect to a touch probe (TP) on a CMM.

## 1. Introduction

The use of commercial-type scanners as non-contact digitizing systems has increased significantly in last years, with a wide range of applications for dimensional metrology and reverse engineering reported [1–3]. Apart from avoiding any influence upon the object to be measured, the main advantage over contact systems is their higher scanning rate, which enables them to capture a great number of points at high speed. Additionally, these systems can be integrated on devices such as coordinate measuring machines (CMM), machine-tools, coordinate measuring arms, specific machines or production systems, which undoubtedly favours their industrial application.

Despite these advantages, current commercial non-contact scanners are usually less accurate than the traditional contact-type methods, since their accuracy depends strongly on the relative position and orientation of the sensor with regard to the digitized part, the configuration parameters of the sensor, the part geometry, the optical properties of surface material, *etc.*

Numerous studies can been found in scientific literature regarding the influence of these and other parameters on laser triangulation digitization. For instance, Vukasinovic *et al.* [4] analysed the influence of incident angle, measurement distance, object colour and reflectivity on the number of points acquired using a laser triangulation scanner. Isheil *et al.* [5] analysed the influence of sensor positioning (distance, incident angle and projected angle) with respect to the measured part. Muralikrishnan *et al.* [6] used a laser triangulation system to measure simple dimensions on prismatic objects and to place bounds on errors derived from different influencing factors such as spot size, part inclination, material or the effect of secondary reflection near the intersection of surfaces. Curless and Levoy [7] found that some of the mentioned errors can be reduced or removed by analysing the time evolution of the light image reflected onto the sensor of the digitizing system. More specifically, Li *et al.* [8] proposed an adaptive dynamic method and measurement compensation of a single-beam laser triangulation for eliminating the effect of the small depth of field in blade inspection.

Feng *et al.* [9] analysed and characterized the digitizing errors of a commercial laser scanner. The objective was to identify the primary scanning process parameters that contributed to the digitizing errors and to establish an empirical relationship to accurately predict the digitizing errors for typical laser scanning operations. In particular, the authors analysed the effect of the scan depth as well as the projected and view angles on process precision. Likewise, they proposed a bilinear model to estimate and correct the effects of these two parameters. Fernández *et al.* [10] and Mahmud *et al.* [11] studied the influence of different parameters on the quality of the points acquired by means of a laser triangulation sensor installed on a CMM in order to determine optimal scanning paths. Gestel *et al.* [12] also described an evaluation test of the performance of a laser profile scanner mounted on a CMM. The authors of this work analysed the influence of distance and scanner orientation with respect to the digitized surface. Godin *et al.* [13] also related the scan depth with changes in measurement distance by the laser scanning system on marble surfaces (translucent and non-homogeneous material). Similarly, they realized that the noise observed in measurements was strongly related to the surface finish.

In view of these studies, it can be stated that laser triangulation is currently a well-established technique, but the performance of other technologies has not been fully described yet. This is the case of Conoscopic Holography (CH).

CH is an interferometric technique based on the double refractive property of birefringent crystals. It was first described by Sirat and Psaltis [14] and patented by Optimet Optical Metrology Ltd. (Jerusalem, Israel). When a polarized monochromatic light ray crosses the crystal, it is divided into two orthogonal polarizations, the ordinary and extraordinary rays, which travel at different speeds through the crystal. The speed of the ordinary ray is constant. However, the speed of the extraordinary ray depends on the angle of incidence. In order to make both rays interfere in the detector plane, two circular polarizers are placed before and after the crystal. The interference pattern obtained in the detector has a radial symmetry, so all the information is contained in one radius. Therefore, given an appropriate calibration, it is possible to calculate the original distance to the light emitting point from the fundamental frequency of one of the signal rays.

Malet and Sirat [15] stated that the performance of a conoscopic system can be described by the quartet of precision depth of field, speed and transverse resolution. Furthermore, many advantages of CH when compared with laser triangulation have been reported by Sirat *et al.* [16], such as better accuracy and repeatability (up to 10 times for a given depth of field), good behaviour for a wide variety of materials (even for translucent materials) and suitability of digitizing sloped surfaces up to 85°. Another practical characteristic is that a single conoscopic sensor can be combined with different lenses to be adapted to various depths of field (0.6 mm up to 120 mm) with accuracy from less than 1 μm up to 60 μm, respectively. Finally, being a collinear system allows for accessing to complex geometries such as holes or narrow cavities, by using simple devices for light redirection.

These characteristics have led CH to be considered in a wide variety of fields, including quality assessment, reverse engineering and in-process inspection. The importance of accuracy becomes an essential target in industrial applications, such as those reviewed by Álvarez *et al.* [17]. This group has successfully applied CH for multiple industrial on-line applications, including sub-micrometric roughness measurements, on-line measurement of high production rate products, surface defect detection in steel at high temperatures and simultaneous inspection of external and internal shape of hollow cylindrical parts.

There are other CH applications far away from the industrial sector, such as those proposed by Spagnolo *et al.* in the legal graphology field. The authors applied a digitizing technique based on CH and a 3D analysis of the acquired data for signature verification. To achieve this objective they proposed methods to determine line crossing order [18] or to study the pressure modulation profile of handwriting [19].

Potential of CH as a valuable alternative to the current well-established technologies (laser triangulation, range sensors or photogrammetry) has led researchers to work on analysing the performance of CH sensors under different scanning conditions.

The ability of CH for digitizing highly sloped surfaces was highlighted by Ko and Park [20] when they compared the capabilities of triangulation, conoscopic holography and interferometry methods for accurate measuring of micro burr geometries formed in micro drilling. They proved that the conoscopic holography method was the most appropriate for measuring small scale burrs (20 μm height and 0.1 mm width). Similar results were reported by Toropov [21] who presented CH as an effective technology for the measurement of burrs above 10 μm. Paviotti *et al.* [22] developed an experimental procedure for analysing the performance of CH sensors when digitizing highly sloped surfaces. They proved that the standard deviation error for the CH sensor remains stable for slope angles up to 60°, but it experiments a sharp increase in the range over 70°.

CH sensor performance is affected by surface properties, as it was highlighted by Lathrop *et al.* [23]. They applied a Conoprobe Mark 3 with a 50 mm objective lens for surface digitization of different types of biological tissues. Experiments were performed for each tissue by adjusting laser power (*P*) and acquisition frequency (*F*) to provide a good quality signal, with a Signal-to-Noise Ratio (*SNR*) over 50%. They found repeatability quite stationary in the whole optical working range (WR) of the lens for each tissue, although different values were met for each material (about σ = 0.01 mm in one case and about σ = 0.15 mm in the other two). Therefore, it can be concluded that the nature of surface material (colour, roughness, texture) has a notable influence on the digitizing quality and, consequently, different adjustment of tuning parameters (*F* and *P*) might be required for different materials.

The use of *SNR* for quality characterization is well-established in CH. Zhu *et al.* [24] employed the *SNR* value provided by the sensor control software, as a quality indicator when digitizing turbine blades. Low *SNR* values (below 70%) were filtered and rejected. Registered measurements revealed part defects which were very difficult to detect with current industrial practices. Lonardo *et al.* [25] applied CH to measure micro and macro geometries of Selective Laser Sintering (SLS) workpieces for zirconia and silica sands. They analysed the influence of *SNR* on three different roughness parameters and found that curves for both materials showed a stationary trend for *SNR* values between 20% and 80%. They also found a dependency of *SNR* with the angle between the laser incidence direction and the surface normal direction. It was reported that *SNR* remains stationary for both materials from 0° (normal direction) up to 75°. Therefore, there was a difference of 10° with regard to the maximum incidence angle of 85° reported by the manufacturer. In the same field, Lombardo *et al.* [26] compared roughness measurement with CH for two identical parts generated by rapid manufacturing techniques, one by stereolithography and the other one by Fused Deposition Modelling (FDM) in a 3D printer. The former showed slightly lower values for parameters *R_a_* and *R_z_* than the latter. Nevertheless, roughness measurements were not compared with other reference values determined by conventional profilometers.

From this review of prior works, it seems clear that *SNR* is assumed as the appropriate indicator for quality assessment when using a CH sensor for digitizing. In most of the cases, CH sensors used in research were adjusted by a combination of *F* and *P* for data acquisition according to instructions provided by the manufacturer. Although *SNR* can range from 0% to 100%, it is desirable to operate under conditions of maximum value of *SNR* when possible, and a minimum *SNR* of 50% is required for acceptable quality [27]. Nevertheless, some works reveal differences when other indicators are used for quality assessment. As it was discussed above, Lathrop [23] used standard deviation and *SNR* as quality indicators, calculated from a collection of measurements for a single point. His work suggests that different materials, digitized under adjusted *F* and *P* for a similar *SNR* value, could provide different values for the standard deviation. In a similar way, Lonardo [25] showed that individual measurements of different points on a material surface present significant variations of the *SNR* value. Actually, *SNR* only reflects the quality of the optical signal, but it has not relation with any metrological parameter regarding the measured object. Therefore, it may be considered whether adjusting working conditions for the highest *SNR* value shall provide the most accurate measurements or not.

No specific work dealing with a systematic adjustment of CH sensor configuration parameters was found. It is assumed that this task relies on the operator skill, and that *SNR* should be the appropriate quality indicator used for this purpose. However, there exist many other parameters that take effect on the reliability of data captured by a CH sensor although there is still little research for analysing this influence.

In the present work, a commercial CH system has been used to analyse the influence of surface location within the WR in combination with the selected values of sensor configuration parameters. The measurements taken by the CH sensor are compared to those acquired by means of a touch probe (TP). Both sensors have been installed on a same CMM.

For the experimental stages, a stepped test specimen has been designed and manufactured in AISI 316 stainless steel by means of wire EDM. Each specimen step has been digitized under different combinations of *F* and *P* and a filtering procedure has made possible to identify those combinations that provide an adequate reconstruction of the specimen. Then, several quality indicators have been defined to compare the behaviour of the CH sensor with respect to the TP. These indicators are calculated considering the surfaces relative location throughout the WR under different combinations of *F* and *P*. Analysis of the results has led to a series of recommendations that will eventually allow for an improvement of scanning quality. The paper structure includes the methodology for the experimental stage, the analysis of results and main conclusions.

## 2. Characteristics of the Conoscopic Holography System

### 2.1. Conoscopic Holography Equipment

The tests described in this study have been performed using an Optimet Conoprobe Mark III conoscopic sensor with a lens of 50 mm focal length and 8 mm of WR. This is a point-type sensor, thus each reading provides the value of distance between the transmitter and the projection of the laser beam on a material surface (spot). The visible light source is a laser diode with a wavelength of 655 nm. Table 1 shows the main characteristics of the sensor provided by the manufacturer.

According to the manufacturer, the *Precision* value was calculated under a procedure that measures dynamically a flat diffusive metallic surface. The minimum sampling step was half of the spot size, and each scan provided the average result of 200 measured points, obtained from a step pattern. In this procedure, only a 50% of the WR was considered. Up to twice less *Precision* would be achieved if the entire WR were considered. Reflective and fine-machined surfaces give approximately twice less *Precision*. For static measurements or small sampling steps (less than half spot size) *Precision* would be up to 1.5 to 2 times less. In any case, the manufacturer does not provide *Precision* information considering the influence of all these effects combined. Then, it can be assumed that the values provided by the manufacturer can only be considered just as reference values, and their validity is constrained to the experimental conditions defined.

In order to achieve a complete digitized surface, a relative displacement between the sensor and the surface is required, which provides a virtual representation of the surface by means of an ordered array of acquired points. The sensor has been integrated into a DEA Swift Coordinate Measuring Machine (CMM), which Maximum Permissible Linear Measuring Tolerance (MPE_E_) and Maximum Permissible Probing Tolerance (MPE_P_) were certified as follows:

(1) $$MPE_{E}=4+4\times 10^{-3}\times L\;[\mu m],\text{being L in}\mathrm{mm}$$

(2) $$MPE_{P}=4\;[\mu m]$$

**Figure 1.**
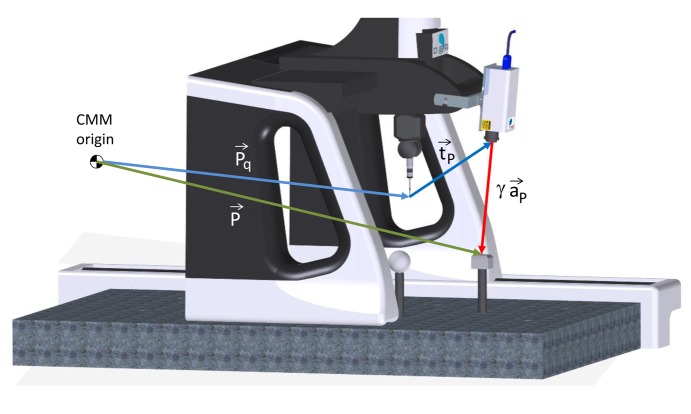
Spatial position of the spot (**P**) related to the CMM origin.

This CMM is operated by means of the measurement and control software PC-DMIS. Volumetric reasons have led to install the sensor on the Y axis of the CMM. This implies that the sensor can be displaced on a plane parallel to the XY reference system, but not in Z direction. In this work, only planar surfaces parallel to the XY plane have been tested, thereby the sensor arrangement in the CMM has not been a constraint for the execution of tests. Considering this arrangement, the spatial position of the spot (**P**) is calculated as a function of three vectors (Figure 1):

– Vector γ·**a_p_**, where γ represents the distance between the spot and the sensor, and **a_p_** is a unit vector along the incident ray direction.
– Vector **t_p_**, which represents the position of the laser emission point with regard to the centre of the CMM touch probe.
– Quill vector **P_q_**, which represents the position of the touch probe with regard to the origin of the CMM coordinate system.

The coordinates of vector **P_q_** are directly provided by the CMM measuring system, whereas vectors **a_p_** and **t_p_** have to be determined by a calibration procedure. In this work, the calibration procedure described by Fernández *et al.* [28] and inspired on the work by Smith [29] has been used.

### 2.2. Configuration Parameters of the CH System

There are two main setting parameters in a CH sensor:

– *Working Frequency* (*F*) represents the data acquisition rate and it can be set up to a maximum of 3,000 Hz. The manufacturer of the sensor recommends using the highest possible *F*, since measurement error can be minimized by better use of averaging filters. Nevertheless, this recommendation can be altered according to the surface optical properties.
– *Power Level* (*P*) represents the value for the laser beam energy and can be set up in a range from 0 to 63.

For a given frequency *F*, the value of power *P* has to be adjusted so that a proper amount of energy reaches the sensor. For a low level of *P*, the amount of light reflected off the surface that reaches the CCD may be insufficient and the quality of the measurement will drop. On the other hand, high values of *P* may yield a saturated signal and the CH sensor will send an out-of-range message, which indicates that the measurement values are not reliable.

## 3. Experimental Method

### 3.1. Test Part

**Figure 2.**
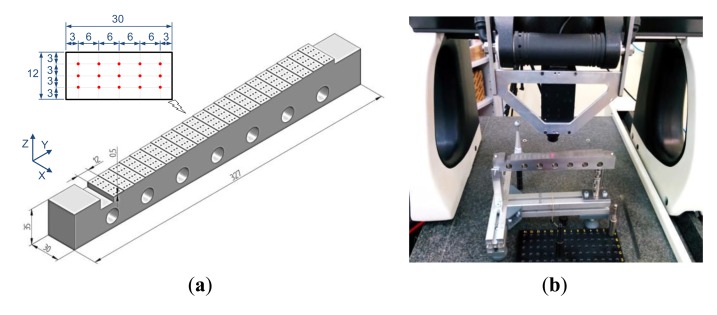
(**a**) General dimensions of the test specimen. (**b**) Detailed view of the test specimen being scanned.

The measurement tests have been conducted on a stepped stainless steel specimen (AISI 316) that has been specially designed for this work. The specimen includes 21 parallel flat steps of 30 × 12 mm of surface (Figure 2a). The nominal height of each step is 0.5 mm, so that a total range of ±5 mm can be analysed with respect to a reference intermediate step located at the middle of the specimen (*datum step*). The specimen was manufactured using a wire EDM process, which minimized the appearance of geometrical distortions.

As any optical-type sensor, quality of measurements carried out with the CH one may be affected by factors such as surface slope. In order to avoid this influence in the experiments, the specimen was attached to a specially designed test bench which allows for locating surfaces of all the steps parallel to the XY reference plane of the CMM (Figure 2b).

### 3.2. Metrological Characterization of the Test Specimen by the Touch Probe

Prior to carrying out the tests with the CH sensor, it was necessary to perform a metrological characterization of the specimen, in order to have reference data to compare with. This was carried out on a CMM by means of a touch probe (TP), inspecting the same points on each step that were measured afterwards with the CH sensor. At this stage, a plane was fitted for each step and two types of distances determined as follows:

– Distances between adjacent steps, denoted as *d^TP^*.
– Distances between each step and the datum step, denoted as *D^TP^*.

### 3.3. Measurement Procedure with the CH Sensor

**Figure 3.**
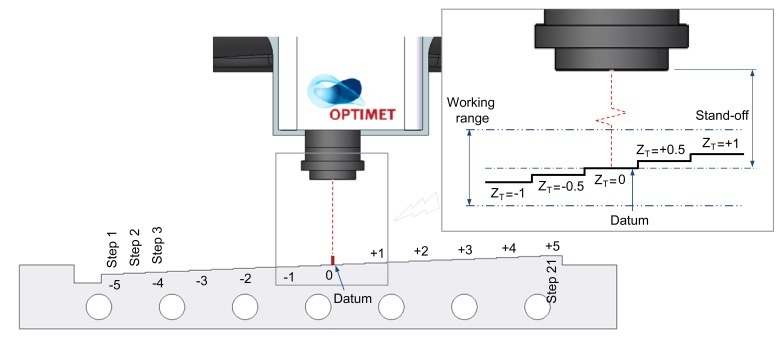
Positioning of the stepped specimen within the WR.

The measurement procedure of the test specimen starts by adjusting the location of the specimen within the WR, so that the datum step of the part (Z_T_ = 0) will coincide at the stand-off distance (Figure 3).

Fifteen points, distributed within a rectangular mesh of 5 × 3 mm, were measured on each step. Five points along *X* direction are captured every 6 mm. Once finished, an increment of 3 mm is performed in *Y* direction for the next measurement routine (Figure 2). This process was repeated for all the steps, from the lowest to the upper. In this work, six levels of frequency *F* have been considered for digitizing ranging from 500 Hz to 3,000 Hz, with increments of 500 Hz. Additionally, twelve levels of power *P* have been used, ranging from 5 to 60 with increments of five units. Subsequently, 72 combinations of *F* and *P* have been tested. The specimen measurement was repeated five times in consecutive days in a laboratory at 20 ± 2 °C. This allowed for performing an analysis using independent data.

Measurements of every single point have been obtained with the sensor completely steady. Up to 100 readings of distance between the sensor and the part surface have been obtained for each point and the average value of them has been considered as the representative distance γ. As it was stated in Section 2.1, the parameter γ allows for determining the coordinates (*X,Y,Z*) of each point with reference to the CMM origin (vector **P**). Besides of distance γ, the sensor provides the value of parameters *SNR* and *Total* met in the measurement. Therefore, the set of values registered for each point may be expressed as follows:

(3) $${\left\{\left(X,Y,Z\right),\text{SNR},\text{Total}\right\}}_{s,i}^{F,P,r}$$

In this expression, *F* and *P* are respectively the frequency and power used in the capture, *r* the number of the experiment (1 to 5), *s* the number of the step considered (1 to 21) and *i* the number of point within the mesh (1 to 15).

### 3.4. Data Filtering

As mentioned previously, *SNR* has been commonly used for describing the quality of a digitized point-cloud. This parameter is calculated by comparison of the peak power value used for the measurement with the whole signal power, which includes signal noise. *SNR* may range from 0% to 100% and it is commonly assumed that the higher the *SNR*, the higher the accuracy of measurement. *SNR* values below 30% indicate non-reliable measurements, whereas values above 50% yield accurate measurement results.

Additionally, the parameter *Total* is provided by the sensor control software. According to the manufacturer, *Total* is proportional to the area limited by the signal envelope and it increases as signal intensity does [27]. Acceptable values for *Total* should be between 2,000 and 16,000.

Considering this, data acquired in the tests have been subjected to a filtering process in order to remove from the study the low quality measurements, as well as those in which a valid register of a point is not obtained. Measurements are rejected in the following situations:

– When they are classified as out-of-range by the capturing software.
– When *SNR* is lower than 50%.
– When value of the parameter *Total* is out of the range 2,000 to 16,000.

After the filtering process, several situations may be observed: non-reconstructed surfaces (if no valid points have been acquired for a particular step), poorly-reconstructed surfaces (if the number of valid points is low for a particular step) and properly-reconstructed surfaces otherwise.

### 3.5. Metrological Characterization of the Test Specimen by the CH Sensor

After the filtering process, the resulting data collected by means of the CH sensor were also processed as it was done for the case of the TP sensor. Thus, distances between adjacent steps as well as distances between each step and the datum were calculated and respectively denoted as *d^CH^* and *D^CH^*.

### 3.6. Quality Indicators

Three quality indicators have been defined in order to characterize the quality of each step geometrical reconstruction:

The first indicator (*n*) has been defined as the number of high quality points resulting from the filtering process for a particular step. Although a maximum of 15 points could be obtained for each single step, the actual value may be lower if signal quality is not good enough. It is assumed that the higher the *n*, the better the fitting of the plane with respect to the digitized points. A minimum of 5 quality points has been established as the limiting condition for a reliable plane reconstruction. Those combinations of F and P which did not provide this minimum number of points in at least one of the 5 repetitions of the experiment have been excluded from the study. A Reliability Area (RA) has been thereafter defined as the reduced set of combinations of *F* and *P* that have always provided an adequate surface reconstruction for the whole specimen.

The second indicator (*X_Δ_*) has been defined as the difference between the distance from each step to the datum calculated by means of CH, and its corresponding reference distance calculated by means of the TP. This indicator can be expressed as follows:

(4) $$X_{\Delta }=D^{CH}-D^{TP}$$

The third indicator (*X_δ_*) has been defined as the difference of the measured distance between adjacent steps calculated by means of CH, and its corresponding reference distance calculated by means of the TP. This indicator can be expressed as follows:

(5) $$X_{\delta }=d^{CH}-d^{TP}$$

Distances measured by means of the TP sensor are not affected by the steps location whereas those measured by the CH are. Thus, indicators *X_Δ_* and *X_δ_* show the influence of each step position within the working range for the CH sensor. High values of these indicators mean that measurements taken by the CH sensor are quite different of those taken by the TP whereas low values mean that the measurements are similar for both sensors.

## 4. Results and Discussion

In this section the behaviour of the three quality indicators (*n*, *X_Δ_*, *X_δ_*) will be analysed considering the values of *F* and *P* and the location of the surface within the WR.

### 4.1. Number of High Quality Points (***n***)

Considering the five trials for each combination of *F* and *P*, the average number of valid points on each step (*n̄*) was calculated as a percentage of the maximum number of digitized points (fifteen points). For instance, Table 2 shows values of (*n̄*) for each step corresponding to a frequency *F* = 3,000 Hz and different values of power *P*.

The combinations of *F* and *P* that guarantee a complete digitization of the specimen within the WR are those whose provide an average number of valid points in all the steps. For the example shown in Table 2, the only valid combinations are (F3000, P20), (F3000, P25), (F3000, P30) and (F3000, P35).

For each of the resulting combinations of *F* and *P*, a global average number of high quality points (*N̄*) was calculated considering all the steps. The final RA is shown in Table 3, where it can be noticed that all the values of *N̄* are higher than 82%. Those combinations of *F* and *P* with the highest values allow for the best geometrical reconstruction.

### 4.2. Difference of Distance X_Δ_

Although *n* indicates the coverage of the geometrical reconstruction, a high value of *n* does not give information about the accuracy of derived measurements (*i.e.*, distance between parallel surfaces). Therefore, it seems necessary to use other metrological indicators. In particular, *X_Δ_* shows the influence of absolute distance measurement between each step and a reference one or datum.

**Figure 4.**
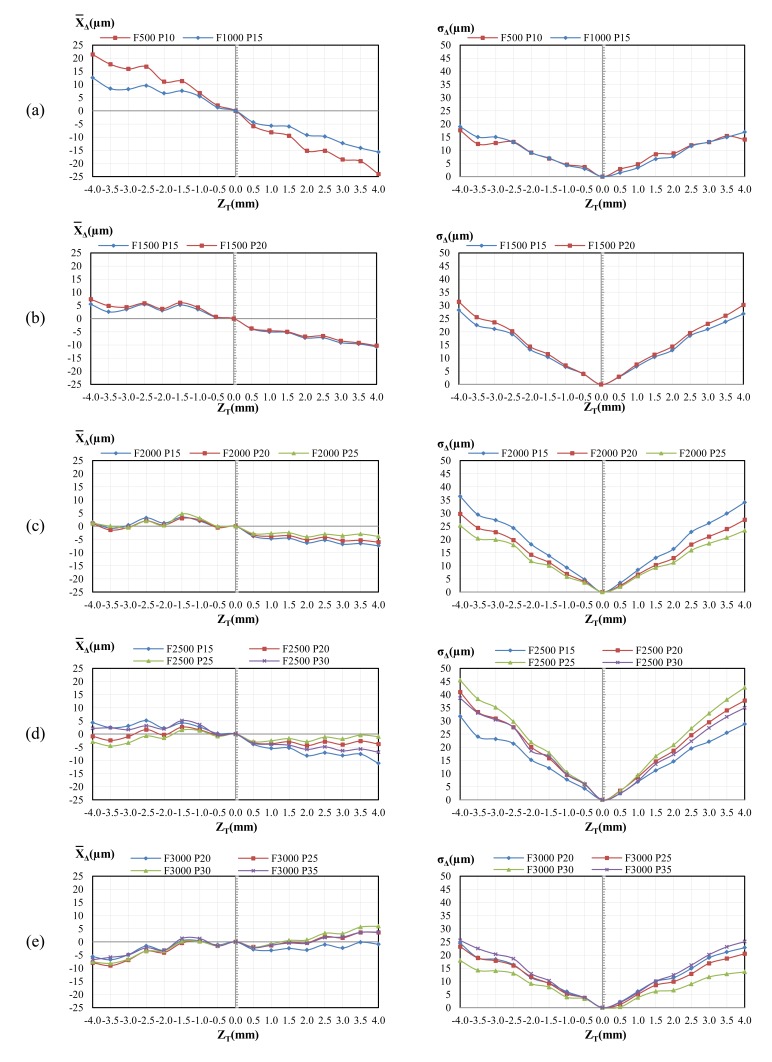
Distribution of *X̄*_Δ_ and *σ*_Δ_ in the working range for all the combinations of *F* and *P* within the RA.

From the five trials, the average value of the indicator (*X̄_Δ_*) and its standard deviation (*σ_Δ_*) were calculated. Figure 4 shows the values obtained for *X̄_Δ_* and *σ_Δ_* for all the combinations of *F* and *P* in the RA previously shown in Table 3. Low values of *X̄_Δ_* indicate that the measurement by the CH sensor is similar to that obtained by the TP. Similarly, small variations of *σ_Δ_* indicate a low dispersion of the indicator *X̄_Δ_* along the five trials.

As shown in Figure 4, the lowest values of *X̄_Δ_* and *σ_Δ_* are obtained for the steps closer to the stand-off. Furthermore, the higher the distance to the stand-off, the greater the value of *X̄_Δ_* and *σ_Δ_*, independently of *F* and *P*. It can be seen that it is difficult to find low values of *X̄_Δ_* and *σ_Δ_* simultaneously. Thus, although *X̄_Δ_* can be reduced to low values varying *F* and *Π*, the evolution of *σ_Δ_* is generally the opposite. This effect can be observed in Figure 4c for the combination (P25, F2000), where *X̄_Δ_* is always lower than 4.69 μm (*Z_T_* = −1.5) while *σ_Δ_* reaches a value of 25.24 μm (*Z_T_* = −4.0). That is, although the average difference is generally low, the dispersion is high and therefore a reliable measurement with the CH sensor is not assured with respect to the TP.

### 4.3. Difference of Distance *X_δ_*

The indicator *X_δ_* has been used with the aim of checking the behaviour of the CH sensor when measuring short distances at different positions of the WR. This indicator represents the difference of distances obtained by the CH sensor and the TP between two adjacent steps.

**Figure 5.**
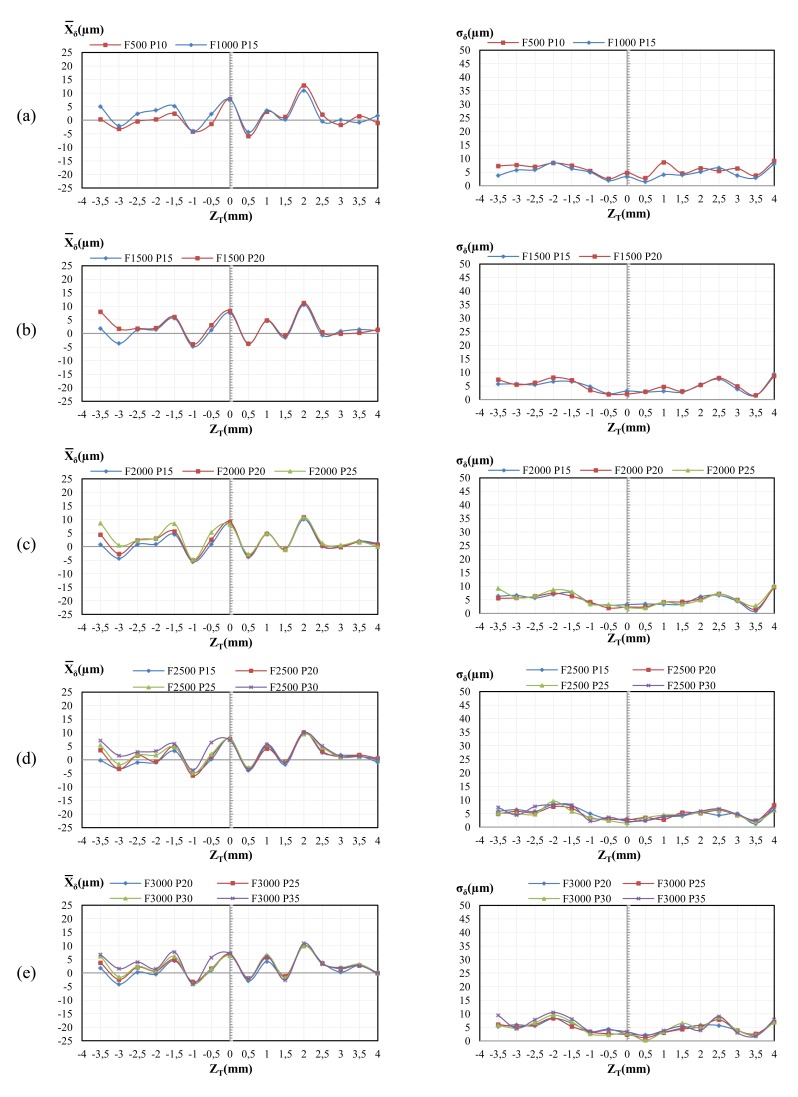
Distribution of *X̄_δ_* and *σ_δ_* in the working range for all the combinations of *F* and *P* within the RA.

From the five trials the average value of the indicator (*X̄_δ_* and its standard deviation (*σ_δ_*) were calculated. Figure 5 shows the values obtained for *X̄_δ_* and *σ_δ_* for all the combinations of *F* and *P* in the RA shown in Table 3. Lower values of *X̄_δ_* indicate that the measurement by the CH sensor is similar to that obtained by the TP. Likewise, small variations of *σ_δ_* indicate a low dispersion of the indicator *X_δ_* along the five trials.

The distribution of *X̄_δ_* values shows a similar behaviour throughout the WR with independence of the selected *F* and *P* combination. Moreover, these values can be positive or negative, which means that sometimes the CH sensor can either overestimate or underestimate the measurements with regard to the TP. In any case, absolute value of *X̄_δ_* is lower than 9 μm in the 95% of cases.

On the other hand, although values of *σ_δ_* also vary randomly, two zones can be distinguished within the WR. For distances between −1.5≤*Z_T_*≤+1.5 values of *σ_δ_* are below 4 μm whereas it varies between 4 and 9 μm for the rest of steps. Although in general these are low values of dispersion they are found once again close to the stand-off. If results are compared, it is found that *X̄_δ_* and *σ_δ_* are similar for all the combinations of *F* and *P*. This reveals that, regardless of the *F* and *P* combination, it can be assured that short distances measured by the CH sensor are similar to those by the TP.

## 5. Conclusions

The present work analyses how the quality of a measurement by a conoscopic holography sensor (CH) is affected by depth of field and configuration parameters. The measurements taken by the CH sensor are compared to those acquired by means of a touch probe (TP). Both sensors have been installed on a same CMM. With this aim, experiments have been performed on an AISI 316 stepped test specimen whose flat surfaces were machined by wire EDM. With the purpose of analysing the sensor behaviour in the whole working range, the tests were performed for all the steps and for different combinations of frequency (*F*) and power (*P*).

Data acquired were subjected to a filtering process in order to remove the combinations of *F* and *P* which led to low quality measurements and which did not allow for a good geometrical reconstruction of the specimen throughout the complete WR. The resulting combinations of this filtering process define the measurement Reliability Area (RA).

In order to know the metrological behaviour of the sensor, three different quality indicators were considered: number of high quality points (*n*) and comparison of distances measured by the CH and the TP (*X*_Δ_ and *X_δ_*). Indicator *n* has permitted us to determine the combinations of *F* and *P* which ensure a high quality reconstruction of all the steps. The grade of quality is represented by the average value of the indicator (*N̄*), which is greater than 82% within the RA in all cases.

Values of indicator *X*_Δ_ depend strongly on the position of the surfaces with respect to the datum since they increase as the surfaces are located farther from the theoretical stand-off distance. In fact, the relationship between the distance difference average (*X̄*_Δ_) and the nominal distance to the stand-off (*Z_T_*) shows an almost-linear behaviour (Figure 4). Nevertheless, the slope of the curve varies with the values of *F* and *P*, so it should be calculated depending on the selected combination. The existence of a constant slope for these curves suggests that *X̄*_Δ_ could be reduced with an appropriate adjustment of *F* and *P*. However, it also has been found that a reduction of *X̄*_Δ_ implies an increase of standard deviation (*σ*_Δ_) so that dispersion rises and this effect is more notorious as the surface is located farther from the stand-off.

On the other hand, indicator *X_δ_* shows almost independent behaviour from the location of each pair of adjacent surfaces within the WR. It also seems not to be affected by the selected combination of *F* and *P*. Dispersion is lower for this indicator than for the previous one, since values for the standard deviation *σ_δ_* usually are below 5 μm. This reflects that calculation of distances between adjacent flat surfaces has similar level of quality throughout the WR for the combinations within the RA. Nevertheless, better results for this parameter are again obtained in locations close to the theoretical stand-off position.

Several conclusions can be highlighted from the study, which are summarized as follows:

– The adjustment criteria based on the values of SNR and Total recommended by the manufacturer should be considered as necessary but not sufficient for guaranteeing good accuracy in the measurements carried out by the CH sensor.
– The recommendation of using a surface located at the stand-off distance for adjusting F and P is not fully adequate since the quality of measurements worsens as distance to that position increases. Thus, there could be situations where surfaces located far from the stand-off distance shall not be properly reconstructed, although good values for SNR and Total parameters have been obtained for surfaces located at the stand-off.
– A high number of digitized points ensures reliable geometrical reconstruction of the surface, but provides no information about the accuracy of the measurement.
– When measuring large distances within the WR, notorious discrepancies are observed between CH and TP sensors. Therefore, measurements of this type are not suggested to be done by means of the CH sensor.
– When short distances are measured, both parameters F and P as well as the position within the WR have no significant influence on the measurements taken by both sensors.

In view of these conclusions, it can be said that metrological behaviour of the CH sensor is more suitable for short distances than for large distances within the WR. For example, this could be applied for comparison of distances with close nominal values. When the sensor is used for measuring larger distances it should be necessary to implement error compensations and adjustment of *F* and *P*.

## Figures and Tables

**Table 1.** Characteristics of the Conoprobe Mark III sensor.

**Property**	**Value**
Dimensions	80 × 180 × 60 mm
Weight	750 g
Measuring speed	875/3,000 Hz
Linearity	0.1%
Working range (WR) (lens 50 mm)	8 mm
Stand-off (lens 50 mm)	42 mm
Static resolution	<0.1 μm
Precision (lens 50 mm)	<6 μm
Reproducibility 1σ (lens 50 mm)	<1 μm
Angular coverage (lens 50 mm)	170°

**Table 2.** Average percentage of valid points (*n̄*) for each step and F = 3,000 Hz.

**Z_T_**	**F3000**												

	**P0**	**P5**	**P10**	**P15**	**P20**	**P25**	**P30**	**P35**	**P40**	**P45**	**P50**	**P55**	**P60**
4.0					100.0	100.0	100.0	98.7	98.7	98.7	97.3	94.7	86.7
3.5				76.0	100.0	100.0	100.0	97.3	97.3	93.3	86.7	78.7	49.3
3.0				89.3	100.0	100.0	100.0	100.0	100.0	100.0	85.3	58.7	
2.5				77.3	100.0	100.0	100.0	100.0	100.0	100.0	98.7	72.0	
2.0				74.7	100.0	100.0	100.0	100.0	96.0	89.3	80.0	54.7	
1.5				98.7	100.0	100.0	100.0	98.7	85.3	66.7			
1.0				98.7	100.0	100.0	100.0	100.0	93.3	77.3	46.7		
0.5				86.7	100.0	100.0	98.7	98.7	98.7	89.3	69.3		
0.0				88.0	100.0	100.0	98.7	96.0	90.7	78.7	60.0	40.0	
−0.5				100.0	100.0	97.3	94.7	88.0	74.7	56.0	38.7		
−1.0				100.0	96.0	81.3	81.3	69.3	48.0				
−1.5				100.0	94.7	94.7	85.3	65.3					
−2.0				100.0	97.3	82.7	72.0	61.3	42.7				
−2.5				100.0	100.0	96.0	88.0	56.0	38.7				
−3.0				100.0	100.0	93.3	90.7	77.3	42.7				
−3.5				100.0	94.7	92.0	80.0	58.7	44.0				
−4.0				100.0	94.7	84.0	66.7	44.0					

*N̄*	-	-	-	-	98.7	95.4	91.5	82.9	-	-	-	-	-

**Table 3.** Average percentage of valid points (*N̄*) for all the steps within the WR.

**F (Hz)**	**P0**	**P5**	**P10**	**P15**	**P20**	**P25**	**P30**	**P35**	**P40**	**P45**	**P50**	**P55**	**P60**
3,000					98.7	95.4	91.5	82.9					
2,500				97.3	97.5	93.9	85.0						
2,000				99.5	95.4	87.5							
1,500				98.2	90.8								
1,000				94.8									
500			95.3										
